# The effect of trauma and alcohol on the relationship between level of cytokines and depression among patients entering psychiatric treatment

**DOI:** 10.1186/s12888-018-1677-z

**Published:** 2018-04-10

**Authors:** Helge Toft, Sudan Prasad Neupane, Jørgen G. Bramness, Terje Tilden, Bruce E. Wampold, Lars Lien

**Affiliations:** 1 Norwegian National Advisory Unit on Concurrent Substance Abuse and Mental Health Disorders, Innlandet Hospital Trust, Post Box 104, Ottestad, N-2381 Brumunddal, Norway; 2 Institute of Clinical Medicine, University of Oslo, Oslo, Norway; 3 Norwegian Center for Addiction Research (SERAF), Institute of Clinical Medicine, University of Oslo, Oslo, Norway; 4 Institute of Clinical Medicine, University of Tromsø, Tromsø, Norway; 5 Research Institute, Modum Bad Psychiatric Center, Vikersund, Norway; 6 University of Wisconsin-Madison, Madison, USA; 7 Department of Public Health, Hedmark University College, Elverum, Norway

**Keywords:** Cytokines, Trauma, Depression, Alcohol, Comorbidity, Immune activation

## Abstract

**Background:**

Depression is associated with immunological responses as reflected by altered levels of circulating cytokines. Alcohol use and trauma may modulate immune activity, and few studies have investigated these factors in depressed patients. We aimed to explore the association between circulating peripheral cytokine levels and degree of depressive symptoms, taking trauma and alcohol into account.

**Methods:**

The study was a cross-sectional assessment of patients at admission to a specialized psychiatric center in Norway. A total of 128 patients were included. Information was gathered using the self-administered questionnaires Beck Depression Inventory-II (BDI-II) and the Alcohol Use Disorders Identification Test (AUDIT), in addition to clinical interviews recording childhood or adult life trauma. Serum levels of the cytokines Interleukin-1β (IL-1β), Interleukin-1 Receptor Antagonist (IL-1RA), Tumor Necrosis Factor-α (TNF-α) and the chemokine Monocyte Chemoattractant Protein-1 (MCP-1) were assessed. A Luminex bead-based multiplex assay was used for cytokine measurements. Patient cytokine levels were compared to those of healthy volunteers by the Mann-Whitney *U* test.

**Results:**

Levels of cytokines did not differ across patients with mild, moderate and severe depression. AUDIT score was not related to cytokine levels, but to level of depression. A history of trauma was related to higher levels of IL-1RA and TNF-α (*p* = 0.048 and *p* = 0.033, respectively), especially among the severely depressed. Serum levels of MCP-1 and TNF-α were significantly higher among psychiatric patients than in healthy volunteers.

**Conclusions:**

Findings indicate that depression was not related to levels of circulating cytokines among patients in treatment, but that traumatized patients had higher levels of IL-1RA and TNF-α than patients without trauma experience. The lack of relationship between cytokine level and depression was evident both in those without and with trauma.

## Background

Patients with major depressive disorder (MDD) are found to have significantly elevated levels of circulating pro-inflammatory cytokines, such as IL-6, TNF-α and IL-1RA [1–3]. There is also some evidence showing that recovery from depressive disorder correlates with return of the inflammatory state to normality [4]. A relationship between immune responses and depression is illustrated by the fact that up to 50% of patients develop clinical depression following high doses of immune therapy with interferon-α (IFN-α) [5]. Also, there is some evidence that elevated cytokine levels may predict depressive illness, suggesting a causal relationship between elevated immune activation and MDD [6, 7].

In addition to MDD, pro-inflammatory cytokines have been found to accompany anxiety disorders such as general anxiety disorder [8, 9], panic disorder and the spectrum of phobias [10] in addition to post-traumatic stress disorder (PTSD) [11]. This may be due to activation of central and peripheral immune cells releasing cytokines, as well as activation of the stress response system of the hypothalamic-pituitary-adrenal (HPA) axis by pro-inflammatory cytokines [12].

One possible confounder for the relationship between cytokines and mental health disorders might be the use of anti-inflammatory drugs, where non-users have higher levels of IL-1β, IL-6, and TNF-α [11]. Other factors that might cause variations are sample characteristics like comorbidity, type of trauma experienced, and time elapsed since the trauma [10, 13]. Such inconsistencies also apply to eating disorders, where previous results are conflicting [14, 15]. This suggests that the relationship between immunological functioning and the broad spectrum of psychiatric disorders should be further examined.

Alcohol use disorder is comorbid in about 30% of patients with major depression, which makes it important to consider alcohol use in investigations of immune changes in depression [16]. Although acute alcohol consumption inhibits immune response, resulting in suppression of the pro-inflammatory cytokines, chronic alcohol use is associated with increased pro-inflammatory cytokine production due to sensitization of immune cells [17, 18]. The resulting dysregulation of the innate immune system may lead to the development of depression [19], and the response may lead to development and progression of depressive disorders among alcoholics [20]. Assessing alcohol dependence to evaluate immune changes in depression patients is therefore critical [21].

Elucidating the role of inflammation in depressed patients in light of previous alcohol use and traumatic life events might provide further insight into the mechanisms of how depressive disorders develop. This could have important translational and clinical implications, such as the development of new therapeutic agents which target the immune system in treatment of major depression. Immunological biomarkers could be used to identify the kind of treatment an individual is likely to benefit from. We aimed to explore the association between circulating peripheral cytokine levels and degree of depressive symptoms, taking trauma and alcohol into account.

## Methods

### Study participants and recruitment procedure

Patients were recruited at admission to Modum Bad, a specialized psychiatric center in Norway. The facility treats patients with long- standing and treatment-resistant trauma, anxiety, eating and depression disorders. Patients with severe self-destructive behavior or psychotic disorders were not eligible for admission. The center does not treat patients with substance abuse disorders (SUD) as such, but many patients have comorbid addiction as part of their problem spectrum. The facility offers group and individual therapies in a 12-week inpatient treatment program. Therapy is paid by public insurance, and patients in work are entitled to sick leave while in treatment. The staff is multidisciplinary, including psychiatrists, psychologists, nurses, art therapists, occupational therapists, social workers and pastoral staff. Data were collected from March 2015 to April 2016. The study was approved by the Norwegian Regional Ethics Committee (REK) prior to data collection (reference number 2014/2189).

Patients were recruited from the following units: Depression, Eating Disorders, Anxiety and Trauma. The patients joined the study in groups of eight at a time. They were given a 15-min presentation about the study during group therapy by the first author on one of the first days of their stay. A written brochure was handed out, explaining the aim of the study and the procedures involved. A written consent form was also distributed to each potential participant. Altogether, 148 (59% of the 249 patients approached) gave their written consent, and one individual withdrew her consent two weeks later. We excluded 19 patients from the data set, due to extreme cytokine levels indicating acute infections. Thus, the present study included baseline data provided by 92 women (72%, mean age 39.04, SD 11.26) and 36 men (28%, mean age 49.06, SD 9.36), giving a total of 128 patients. The 102 patients who did not participate consisted of 82 women (80%, mean age 35.77, SD 11.83) and 21 men (21%, mean age 44.52, SD 8.58).

### Healthy volunteers group

As a control group, we utilized data from an experimental study with healthy volunteers performed by our group [22]. Sampling procedures are detailed in the original study. In short, healthy volunteers were recruited through an advertisement in the Correctional Service Staff Academy in Oslo, Norway. The volunteers were 20 males aged from 20 to 45 (mean age 28.84, SD 5.3) who all had previous experience of high-dose alcohol drinking. Exclusion criteria were having any significant medical illness, alcohol or other substance use disorders, or metabolic disorders. Demographic information was recorded, the AUDIT questionnaire was answered, and cytokines were analyzed by venous blood collection. The blood was collected at 7 am following an overnight fasting. One volunteer was excluded due to an extreme blood level of TNF-α. For the experimental study, an inclusion criterion was some experience of high-dose alcohol drinking but current non-dependence on alcohol. Thus, an inclusion maximum was set to 15 on the AUDIT. As this volunteer group differed in age and gender from the patients in our clinical study, we selected the youngest male patients from our study to be compared to this healthy volunteer sample with regard to cytokine levels.

### Methods

All patients were interviewed by trained psychologists or psychiatrists using the Mini-International Neuropsychiatric Interview (MINI) [23]. The measurements were conducted during the first week of admission. The MINI interview results in a diagnosis in the 10th revision of the International Classification of Diseases and Related Health Problems (ICD-10). A combination of psychometric questionnaires and clinical judgment was also taken into account in assessing disorders. There were 54 patients with only one disorder, and 59 with two or more disorders. There were 15 patients with missing diagnosis and 14 with missing trauma status due to staff failing to record the patient’s disorder. Disorders within F30–39 were treated as one variable of mood disorders. Disorders within the range of F40–49 were merged to one variable of anxiety disorders. Disorders within F50.0-F50.9 were merged to one variable of eating disorders.

In addition, the patients completed various self-report questionnaires on a computer or digital tablet. The following questionnaires were included:

The 21-item Beck Depression Inventory (BDI-II). The BDI-II [24] was administered by the therapists to assess the level of depressive symptoms during the two weeks prior to the interview. The Norwegian validated version was used [25, 26]. Each of the 21 items is scored from 0 to 3. Based on the average score, we categorized the answers into three levels of depression severity: Minimal and mild depression (score 0–18), moderate (score 19–29), and severe (score 30–63).

The Alcohol Use Disorder Identification Test (AUDIT). This is a 10-item screening test designed to identify harmful, hazardous or possible alcohol dependence the last 12 months. The AUDIT has been proven able to detect DSM-IV alcohol dependence and DSM-IV alcohol use disorder (AUD) when compared with semi-structured clinical interviews [27]. Some examples of questions are: “How often do you have a drink containing alcohol?” and “How many drinks containing alcohol do you have on a typical day when you are drinking?” The scores range from 0 to 4. In these examples, a score of 0 refers to “never” and “1–2 drinks”, respectively. A higher score refers to a more severe drinking pattern, and in these two examples, a score of 4 means “4 times a week” and “10 or more drinks”. The cutoff scores for harmful or hazardous drinking were set at 8 for men and 6 for women [28].

Trauma history was recorded by therapists at the facility as part of clinical history taking. There were five questions on trauma exposure: 1) Has the patient been exposed to sexual assaults in childhood? 2) Has the patient been exposed to physical abuse in childhood? 3) Has the patient during childhood experienced other traumatic events which have led to serious problems later in life? 4) Has the patient been exposed to sexual assaults or abuse in adulthood (after 18 years of age)? 5) Has the patient in adulthood experienced other traumatic events which led to serious problems later?

### Blood collection and serum preparation

The blood samples were taken in the laboratory at Modum Bad between 8 and 9 am. One of the groups from the Depression Unit had their blood samples taken between 12 and 3 pm. The blood samples were collected in Vacuette 8 ml serum tubes, which were immediately turned upside down about 8–10 times. They were then set to rest in a blood tube stand for between 30 min and one hour before being centrifuged in a Kubota 2420 swing-out centrifuge at room temperature. The centrifuging procedure was set to 10 min, and the centrifuge achieved a rotation power of 1917 g. The separated serum was drawn from the Vacuette tubes with a 1 ml single use pipette into two 2 ml Nunc tubes. The samples were then stored in a − 80 degrees Celsius freezer until assay.

### Cytokine measurements

All samples were thawed on ice, vortexed, and then spun down a tube with 250 μl serum at 14,000×g for 10 min at 4 °C, before dilution (1 + 4) and further processing. The following cytokines were assessed: IL-1β, IL-1RA, IL-6, IL-10, IL-17A, IFN-y, MCP-1 and TNF-α. These cytokines were selected based on the available literature on the neuroimmune correlates of psychiatric disorders. We present cytokines that were within the detectable range, which were IL-1β, IL-1RA, MCP-1 and TNF-α. Cytokine measurements were performed using Bio-Plex xMAP technology (Bio-Rad, Austin, Texas, USA) with a Luminex IS 100 instrument (Bio-Rad, Hercules, California, USA), powered using Bio-Plex Manager (version 6.0.1) software. Multiplex bead-based technologies such as Luminex allow detection and quantification of multiple cytokines with good efficiency, speed and dynamic range at reasonable cost. The assay was performed according to the manufacturer’s instructions, but an additional standard point was included. To achieve a more reliable result, individual sets of samples from patients were run in the same assay, all samples were assayed in duplicate and a magnetic plate washer was used during assay set up. The StatLIA software package (version 3.2, Brendan Scientific, Carlsbad, California, USA), incorporating a weighted, five-parameter logistic curve-fitting method, was used to calculate sample cytokine concentrations. Longitudinal controls were used in order to validate inter-assay variation: IL-1β (18.1), IL-1RA (10.2), MCP-1 (6.7) and TNF-α (7.4). Numbers in parentheses are inter-assay coefficients of variability (CV), where a lower number is better. Any number below 21 is considered acceptable. The mean inter-assay CV for all blood sample plates was 10.4%. The serum levels were measured in picograms per milliliter (pg/ml). The minimum detectable values were 0.01 pg/ml for IL-1β, 3 pg/ml for IL-1RA, 0.76 pg/ml for MCP-1, and 0.02 pg/ml for TNF-α.

### Statistical analysis

The statistical package SPSS version 23 for Windows (SPSS Inc., Chicago IL, USA) was used for the statistical analysis. One patient failed to fill out the AUDIT questionnaire, and was treated as missing and excluded from the analysis. Descriptive statistics were used to present the cytokine values. The values were rather skewed, and therefore presented by medians and 25/75 percentiles. We attempted to normalize the skewed cytokine data by log transformation. However, the Kolmogorov-Smirnov test of normality remained significant, and we therefore did not explore this approach further. Nonetheless, we explored the analyses with and without log-transformed data, which remained unchanged. Undetectable cytokine levels were imputed with 1% of the mean value. In the patients, 64 (50.39%) imputations of the cytokine IL-1β were conducted, 1 (0.9%) of IL-1RA, 9 (7.09%) of MCP-1, and 53 (41.73% of TNF-α. In the healthy volunteers, 6 (31.6%) imputations of IL-1β were conducted, none of IL-1RA, 5 (26.3%) of MCP-1, and 15 (79%) of TNF-α. These undetectable cytokine levels were above zero, but under the detectable limit, leaving imputation as a way of presenting undetectable levels in close resemblance to their actual levels. Patients with cytokine levels above the 95th percentile were defined as outliers, and removed from the material, thus reducing the potential distortion from any somatic inflammatory diseases. For the continuous variable of age, Spearman’s rho correlation coefficient was used. The significance level between the cytokines and categorical variables was calculated by the Mann-Whitney *U*-test and by the Kruskal-Wallis one-way ANOVA. Pearson’s chi-square was used for calculating the significance level between the categorical variables. Finally, a comparison of mean cytokine values between patients and healthy volunteers was made. Group differences in the levels of cytokines between the patients and healthy volunteers were calculated using the Mann-Whitney *U* test, and *p*-values for the tests are reported. All tests were two-tailed with statistical significance set at the 5% level. Some of the group-wise comparisons may have lacked statistical power due to small group sizes, raising the possibility of type II error.

## Results

Table 1 presents demography, medication, trauma history, and diagnosis across the BDI-II scores categorized into three different levels. The only difference between these three levels of depression was the AUDIT scores, where the higher score was in those with moderate depression (*p* = 0.009). The use of anti-inflammatory and anti-depressive medication was positively associated with depression severity. A positive history of trauma was most frequent in the severely depressed patients. Patients classified under the main diagnostic groups (mood/anxiety/eating/trauma) had a uniform distribution of depression severity.

**Table 1:** Clinical characteristics of the included patients according to level of depression. Depression severity was measured by the Beck Depression Inventory-II (BDI-II)

Variable		Minimal or mild depression^a^		Moderate depression		Severe depression		
		*n* = 28 (21.9%)	% / SD	*n* = 42 (32.8%)	% / SD	*n* = 58 (45.3%)	% / SD	Sig.^b^
Demography								
Women	n (%)	20	71.4	28	66.7	44	75.9	0.600
Age (years)	Mean (SD)	39.4	10.8	44.2	12.0	41.3	11.7	0.210
Alcohol use								
AUDIT score^c^	Mean (SD)	4.70	4.1	6.7	5.4	3.7	4.3	*0.009* ^*d*^
Medication								
Anti-inflammatory drug (any)	n (%)	5	17.9	5	11.9	9	15.5	0.775
Antidepressants (any)	n (%)	9	32.1	12	28.6	22	37.9	0.609
History of trauma								
Childhood trauma	n (%)	15	62.5	23	62.2	25	48.1	0.316
Adult trauma	n (%)	16	66.7	16	43.2	28	53.8	0.199
Any trauma	n (%)	19	79.2	26	70.3	39	73.6	0.743
Main diagnosis^e^								
Mood disorder	n (%)	8	34.8	14	36.8	10	19.2	0.139
Anxiety disorder	n (%)	12	52.2	17	44.7	26	50	0.825
Eating disorder	n (%)	3	13.6	5	13.2	14	26.9	0.181
MDD (ICD-10, F33)^f^	n (%)	3	10.7	5	11.9	3	5.2	0.447

Notes: ^a^14 of these patients scored 13 or less in the BDI-II, indicating that they were not depressed, and were classified with minimal depression. ^b^Significance for categorical variables was tested with Pearson’s chi-square test, and for continuous variables with Kruskal-Wallis one-way ANOVA. ^c^AUDIT: Alcohol Use Disorder Identification Test. ^d^AUDIT score was different across the levels of BDI-II. ^e^Main diagnosis: Mood disorders: F30-F34, Anxiety disorders: F40-F44, Eating disorders: F50.0-F50.3. ^f^MDD: Major Depression Disorder, as classified by the ICD-10

Table 2 shows the distribution of cytokine values according to demography, distress, medication, trauma and diagnostic groups. Levels of IL-1β were significantly higher in the group with mood disorder and anxiety disorder than in those without (*p* = 0.037 and *p* = 0.008, respectively). IL-1RA and TNF-α were significantly higher in patients with a history of trauma than in those without trauma experience (*p* = 0.048 and *p* = 0.033, respectively). Participants scoring above cutoff in the AUDIT had higher values of all measured cytokines; however, the differences were not statistically significant.

**Table 2:** Median levels (25th and 75th percentile) of peripheral circulating cytokines according to clinical characteristics of the included patients

Variable		IL-1β (pg/mL)		IL-1RA (pg/mL)		TNF-α (pg/mL)		MCP-1 (pg/mL)	
		Median (25th, 75th percentile)	Sig.^a^	Median (25th, 75th percentile)	Sig.^a^	Median (25th, 75th percentile)	Sig.^a^	Median (25th, 75th percentile)	Sig.^a^
Demography									
Men	*n* = 36	0.003 (0.003, 0.130)	0.604	26.2 (17.8, 46.1)	0.804	0.080 (0.046, 1.390)	0.346	27.7 (15.3, 43.7)	0.069
Women	*n* = 91	0.030 (0.003, 0.160)		27.5 (17.4, 40.3)		0.420 (0.046, 2.450)		21.1 (11.5, 32.6)	
Age		0.033	0.716	0.141	0.114	0.068	0.450	0.027	0.762
Psychometrics									
AUDIT (under cutoff score)	*n* = 95	0.003 (0.003, 0.160)	0.730	24.8 (16.5, 42.5)	0.342	0.420 (0.046, 1.930)	0.768	20.8 (11.4, 33.1)	0.497
AUDIT (over cutoff score)	*n* = 32	0.050 (0.003, 0.128)		28.1 (20.5, 39.0)		0.420 (0.046, 2.393)		24.8 (10.4, 40.3)	
BDI-2 (minimal and mild depression)	*n* = 27	0.003 (0.003, 0.200)	0.840	24.0 (15.4, 41.1)	0.564	0.420 (0.046, 2.450)	0.973	23.7 (14.4, 40.4)	0.277
BDI-2 (moderate and severe depression)	*n* = 100	0.025 (0.003, 0.130)		27.5 (17.5, 42.9)		0.245 (0.046, 2.108)		21.9 (12.4, 34.1)	
Medication									
Anti-inflammatory drugs (no)	*n* = 108	0.003 (0.003, 0.130)	*0.050* ^*b*^	27.5 (17.4, 42.8)	0.634	0.180 (0.046, 2.008)	0.818	22.9 (13.2, 34.3)	0.095
Anti-inflammatory drugs (yes)	*n* = 19	0.090 (0.003, 0.300)		24.8 (17.5, 34.3)		1.020 (0.060, 3.410)		22.2 (10.7, 38.6)	
Antidepressants (no)	*n* = 85	0.040 (0.003, 0.160)	0.510	27.5 (17.0, 42.5)	0.654	0.420 (0.046, 2.455)	0.370	24.3 (14.8, 37.9)	0.126
Antidepressants (yes)	n = 42	0.003 (0.003, 0.133)		26.7 (19.6, 41.0)		0.185 (0.046, 1.250)		19.3 (10.6, 33.1)	
History of trauma									
Childhood trauma (no)	*n* = 50	0.003 (0.003, 0.123)	0.379	24.5 (16.3, 39.4)	0.215	0.090 (0.046, 1.060)	0.060	23.8 (14.7, 37.7)	0.576
Childhood trauma (yes)	*n* = 62	0.012 (0.003, 0.218)		28.1 (19.9, 41.3)		0.920 (0.046, 3.068)		22.9 (10.4, 36.9)	
Adult trauma (no)	*n* = 53	0.003 (0.003, 0.100)	0.235	22.8 (17.0, 35.5)	0.097	0.310 (0.046, 2.095)	0.786	23.3 (13.4, 33.1)	0.438
Adult trauma (yes)	*n* = 59	0.060 (0.003, 0.170)		28.3 (18.7, 42.3)		0.420 (0.046, 2.450)		24.9 (10.7, 43.0)	
Any trauma (no)	*n* = 30	0.003 (0.003, 0.100)	0.154	21.7 (14.8, 30.7)	*0.048* ^*c*^	0.070 (0.046, 0.775)	*0.033* ^*d*^	23.5 (16.9, 32.7)	0.838
Any trauma (yes)	*n* = 83	0.040 (0.003, 0.190)		28.4 (18.7, 42.1)		0.920 (0.046, 2.900)		23.7 (10.6, 38.6)	
Main diagnosis^e^									
Mood disorder (no)	*n* = 80	0.060 (0.003, 0.200)	*0.037* ^*f*^	27.1 (17.3, 41.7)	0.982	0.390 (0.046, 2.450)	0.634	22.8 (11.6, 38.4)	0.829
Mood disorder (yes)	n = 32	0.003 (0.003, 0.060)		26.9 (20.4, 39.3)		0.310 (0.046, 2.045)		24.1 (14.1, 33.4)	
Anxiety disorder (no)	*n* = 55	0.003 (0.003, 0.090)	*0.008* ^*g*^	25.5 (17.5, 37.6)	0.325	0.060 (0.046, 1.780)	0.111	23.8 (13.9, 33.1)	0.740
Anxiety disorder (yes)	*n* = 57	0.090 (0.003, 0.270)		29.3 (18.7, 44.1)		0.920 (0.046, 3.340)		23.3 (10.0, 43.0)	
Eating disorder (no)	n = 91	0.003 (0.003, 0.160)	0.429	28.1 (18.7, 42.1)	0.171	0.420 (0.046, 2.460)	0.561	23.7 (13.2, 38.6)	0.414
Eating disorder (yes)	*n* = 21	0.003 (0.003, 0.115)		22.3 (15.7, 36.0)		0.100 (0.046, 1.905)		22.2 (11.8, 31.3)	

Notes: ^a^Significance levels for categorical variables were tested with the Mann-Whitney *U* test and for continuous variables with Spearman’s rho correlation. ^b^Patients using anti-inflammatory drugs had higher levels of IL-1β. Exact p-value: 0.0498. ^c + d^Patients with trauma history had higher levels of IL-1RA and TNF-α. ^e^Main diagnosis: Mood disorders: F30-F34, Anxiety disorders: F40-F44, Eating disorders: F50.0-F50.3. ^f^Patients with mood disorders had higher levels of IL-1β. ^g^Patients with anxiety disorders had higher levels of IL-1β

Table 3 displays mean cytokine values from the 19 male, healthy volunteers and 19 matched patients from our clinical study. All levels were higher in the patient group than in the healthy volunteers, with MCP-1 and TNF-α reaching statistical significance (*p* = 0.012 and *p* <  0.001, respectively). The selected patient group was older than the healthy volunteers group (*p* < 0.001). We also performed analyses of a patient cohort consisting of all patients compared to the healthy volunteers, and of all 36 male patients compared to the healthy volunteers. However, these different approaches did not change the results significantly, the findings being consistent across male and female patients, as well as between the whole sample and the healthy volunteers sample.

**Table 3:** Mean age (SD) and mean levels (SD) of peripheral circulating cytokines in healthy volunteers and a subgroup of matched patients

Variable		Matched patients	Healthy volunteers	Sig.^a^
		n = 19	n = 19	
Age	(years)	42.5 (7.9)	28.8 (5.4)	*<0.001* ^*b*^
Il-1β	(picograms/ml)	0.15 (0.31)	0.13 (0.19)	0.740
Il-1RA	(picograms/ml)	36.3 (33.0)	35.4 (19.5)	0.385
TNF-α	(picograms/ml)	2.67 (6.67)	0.82 (2.75)	*< 0.001* ^*c*^
MCP-1	(picograms/ml)	26.8 (21.4)	15.8 (15.5)	*0.012* ^*d*^

Note: ^a^Significance levels were tested by the Mann-Whitney *U* test. ^b^Patients were older than the healthy volunteers. ^c^^+^^d^ Levels of TNF-α and MCP-1 were higher in patients

Both healthy volunteers and matched patients were male

**Fig. 1:**
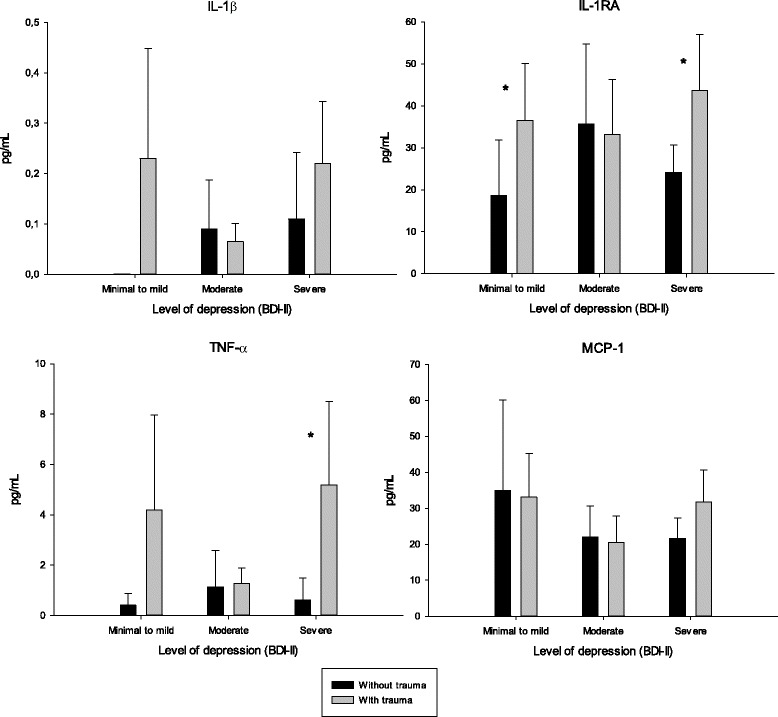
Distribution of cytokine levels (mean, upper bound of 95% CI) in depression groups for non-traumatized vs. traumatized patients (any trauma; childhood or adult) grouped by the Beck Depression Inventory-II. An asterisk (*) denotes a significant difference between groups (Mann-Whitney *U*-test) (*p* <  0.05)

**Fig. 2:**
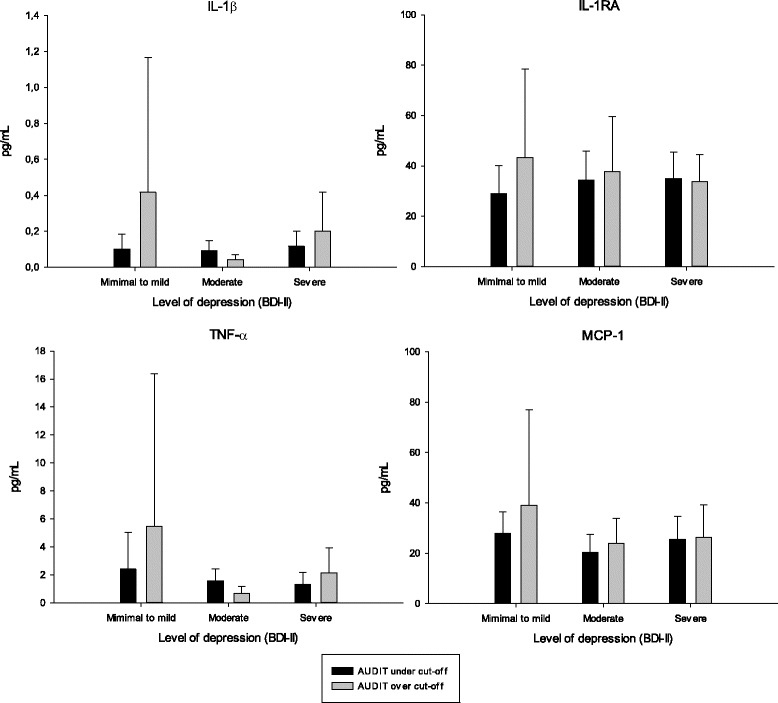
Distribution of cytokine levels (mean, upper bound of 95% CI) in depression groups for patients scoring below vs. above clinical cutoff for AUDIT, grouped by Beck Depression Inventory-II

To investigate possible effects of trauma history or alcohol use on the relationship between cytokine levels and level of depression as quantified by the BDI-II, we stratified the material into non-traumatized and traumatized patients (Fig. 1) and those scoring below or above clinical cutoff on AUDIT (Fig. 2). As depicted in Fig. 1, patients who were categorized in the minimal or mild depression group, as well as in the severe depression group, had significantly different levels of cytokine IL-1RA when stratified on trauma and no trauma experience (*p* = 0.046 and *p* = 0.047, respectively). This was also true for levels of TNF-α with the same strata in the severe depression group (*p* = 0.029). There were no significant relationships between cytokine levels and depression levels in the two strata of patients below and above AUDIT score.

## Discussion

Patients with mood disorder and anxiety disorder had higher levels of IL-1β, but we found no systematic relationship between level of depression and level of the measured cytokines. Patients reporting trauma had higher levels of the cytokines IL-1RA and TNF-α. We found no significant relationship between cytokines and AUDIT scores.

The observed lack of relationship between cytokine levels and level of depression may be because patients at this high-threshold facility all have increased levels of cytokines at admission. They represent a treatment-resistant population that has suffered for years with mental illness and received mental health treatment with no or little effect. A consequence may be that the immune system is chronically activated. However, despite not reaching statistical significance, mean and median cytokine levels were generally higher in groups who scored high on depression. The use of both antidepressants and anti-inflammatory drugs has been reported to distort the immune response, which may have contributed to the non-significant findings [29].

We found a relationship between patients suffering childhood or adulthood trauma and higher cytokine levels, represented by IL-1RA and TNF-α being significantly elevated, but we found no links between childhood trauma or adulthood trauma and depression level. This led us to perform a stratified analysis investigating non-traumatized and traumatized patients separately. In these strata, we found a difference in levels of IL-1RA in the minimal to mild depression group, and in levels of IL-1RA and TNF-α in the severe depression group. Stress-induced depression may follow inhibited glucocorticoid release as a result of a chronically activated HPA axis, a sign of inflammation [30]. In traumatized or maltreated children, insufficient glucocorticoid signaling may potentially lead to an unrestrained state of inflammation as adults, rendering them vulnerable to depression [31]. Studies report that childhood maltreatment is a predictor of elevated levels of inflammatory markers in adulthood, and such individuals are more susceptible to developing depression [32]. Also, adult experiences of trauma may lead to the development of chronic stress and thus elevated pro-inflammatory cytokines [33].

The elevated IL-1RA level in the traumatized patients may be a result of the initial inflammatory response being countered by a down-regulated inflammatory response [34, 35], limiting the pro-inflammatory effects of IL-1 [36]. Previous studies found elevated levels of IL-1β [11, 37, 38] and TNF-α [11, 39], while others found no such association of IL-1β [39], TNF-α [40] or other neuroimmune biomarkers. One possible explanation for these mixed results may be that different kinds of trauma yield different immunologic responses. For instance, whether the trauma was experienced as a child or as an adult is a relevant factor [13]. The HPA axis and brain in childhood are immature and developing, and the adaptive response to maltreatment has long-lasting effects on the stress response system. As a consequence, some individuals develop a persistent vulnerability to stressors. This maladaptive vulnerability accompanies them in adult life, potentially making such individuals more susceptible to developing depression or anxiety disorders [41, 42].

Score above cutoff on the AUDIT questionnaire, possibly indicating alcohol use problems, was related to level of depression. However, we found no relationship between levels of cytokines and AUDIT. This differed from our previous cross-sectional study in Nepal, where we found increased pro-inflammatory cytokines in AUD (alcohol use disorder) patients with comorbid MDD, but not with PTSD [43, 44]. In line with the findings in that study, we investigated whether there were different relationships between cytokine levels and levels of depression in those with or without an alcohol problem, but analyzing the data in this way by stratifying according to AUDIT score did not reveal any such effect in the present study. The literature suggests that low levels of drinking might dampen an immunological response, while high levels may increase circulating cytokines [18]. In the current study, patients scored quite low on the AUDIT, indicating low levels of alcohol problems. This might explain the lack of relationship between cytokines and AUDIT score.

When healthy volunteers were compared to a group of matched patients, the patients were found to have higher levels of the cytokines TNF-α and MCP-1. Although some studies have found MCP-1 to be lower in patients with MDD [45], our findings of higher levels of TNF-α and MCP-1 in the patient group add to several studies showing a correlation between psychiatric disorders and elevated pro-inflammatory cytokines [46]. This general heightened cytokine level among the patients could contribute to masking differences in cytokines between different levels of depression in the patients.

There are limitations to this study. The 19 healthy volunteers were younger than the 19 youngest matched male patients. Aging is associated with higher risk of comorbidity, and these factors may result in increased levels of TNF-α and IL-1 [47]. The healthy volunteers were male only, which was unfortunate since the majority of the patients were female. Unlike the healthy volunteers, the patients were not fasting when the blood samples were taken. Eating three meals a day versus one meal a day has been shown to elevate levels of TNF-α and MCP-1, suggesting eating habits could mediate inflammatory pathways [48]. The Body Mass Index (BMI) of the patients was not assessed in this study. Studies have shown the levels of pro-inflammatory cytokines to be higher in obese people (BMI > 30 kg/m2). Adipocytes are known to release circulating cytokines, notably TNF-α, IL-1β and IL-6 [49], and one cannot exclude the possibility of BMI confounding the cytokine levels. Trauma history was recorded during clinical interviews using five standardized questions regarding child and/or adult physical or sexual abuse. These questions are less valid than validated trauma questionnaires, but provide face validity since they were discussed with each patient by trained staff. Furthermore, the study is cross-sectional and should be followed by longitudinal studies. We decided to report the results with uncorrected *p*-values. Using Bonferroni correction or similar would be an overly conservative approach when conducting multiple tests in an exploratory study. Consequently, there is also a risk of type I errors. Against this background, one should interpret the results with caution. Despite these limitations, this study fills gaps in the literature by elucidating cytokine levels across trauma and depression symptoms. Finally, there is a risk of type II errors when analyzing such small samples.

## Conclusions

Patients with a history of trauma had higher levels of the cytokines IL-1RA and TNF-α. There were no associations between cytokine levels and depression severity, even when analyzing in sub-groups stratified according to variables that themselves were related to cytokine level like trauma and alcohol use.

## Abbreviations

AUD: Alcohol Use Disorder
AUDIT: Alcohol Use Disorders Identification Test
BDI-II: Beck Depression Inventory-II
HPA axis: Hypothalamic-pituitary-adrenal axis
ICD-10: International Classification of Diseases and Related Health Problems 10th revision
IL-1RA: Interleukin-1 Receptor Antagonist
IL-1β: Interleukin-1 beta
IL-6: Interleukin-6
MCP-1: Monocyte Chemoattractant Protein-1
MDD: Major Depression Disorder
MINI: Mini-international Neuropsychiatric Interview
PTSD: Post-traumatic Stress Disorder
TNF-α: Tumor Necrosis Factor-alpha
